# APOBEC3C‐Mediated NF‐κB Activation Promotes Malignant Progression of Gliomas

**DOI:** 10.1002/iid3.70224

**Published:** 2025-07-24

**Authors:** Chao Zhang, Tao Yang, Yuhang Tang, Dong Yu, Shiqiang Hou, Ning Lin, Qun Li

**Affiliations:** ^1^ Department of Neurosurgery The Affiliated Chuzhou Hospital of Anhui Medical University, The First People's Hospital of Chuzhou Chuzhou China; ^2^ Health Examination Center The Affiliated Chuzhou Hospital of Anhui Medical University, The First People's Hospital of Chuzhou Chuzhou China

**Keywords:** APOBEC3C, glioma, NF‐κB, prognosis

## Abstract

**Objective:**

To analyze the clinicopathological features, immunological characteristics, and prognostic value of APOBEC3C in gliomas, and to verify the specific mechanism by which it mediates the malignant progression of gliomas.

**Methods:**

mRNA‐seq data from 693 glioma patients in the CGGA database and 697 glioma patients in the TCGA were analyzed, respectively. In addition, single‐cell sequencing data were obtained from the CGGA database. Bioinformatics methods were applied to reveal the possible mechanisms of APOBEC3C‐mediated malignant progression of gliomas. Moreover, western blot, transwell, and cell‐scratch assays were used to explore the potential mechanisms of APOBEC3C‐mediated glioma invasion and migration.

**Results:**

APOBEC3C was enriched in malignant glioma subtypes and was a potential biomarker for mesenchymal subtypes in glioma patients. It was closely associated with glioma inflammation and immunity. Additionally, APOBEC3C is a potential independent prognostic factor for glioma, and inhibition of APOBEC3C expression can suppress the EMT process in glioma cells through the NF‐κB signaling pathway.

**Conclusion:**

APOBEC3C is a potential biomarker for glioma patients. It is closely related to the clinicopathology of glioma and may be a potential immunotherapy target for glioma patients.

**Abbreviations**
CGGA: The Chinese Glioma Genome Atlas
EMT: epithelial–mesenchymal transition
GBM: glioblastoma multiforme
GO: Gene Ontology
K–M: Kaplan–Meier
KEGG: Kyoto Encyclopedia of Genes and Genomes
LGG: low‐grade gliomas
OS: overall survival
ROC: receiver operating characteristic
TCGA: The Cancer Genome Atlas
TME: tumor microenvironment
Tregs: regulatory T cells

## 1 Introduction

Among primary malignant brain tumors, gliomas are the most common. Originating from glial and neuronal cells, they account for the majority of intracranial tumors [1]. Gliomas are much more invasive and prone to recurrence compared to other intracranial tumors. Based on molecular features, gliomas are classified into proneural, neural, classical, and mesenchymal subtypes [2]. Currently, the treatment for gliomas mainly consists of surgical resection with maximum safety margins. Despite postoperative chemoradiotherapy, the prognosis for patients remains poor, with a median survival of only 15 months [3]. Therefore, there is an urgent need to understand the molecular mechanisms underlying the malignant progression of gliomas.

Glioma patients have a poor prognosis with conventional treatments such as surgery, radiotherapy, and chemotherapy. As a novel treatment strategy, cancer immunotherapy can activate the immune system to target cancer cells [4]. In the immune response against glioblastoma multiforme (GBM), immune checkpoints regulate T‐cell activity to stimulate the immune system [5]. High expression of immune checkpoints, such as PD‐L1 (PDCD1LG2), TIM‐3 (HAVCR2), and CTLA‐4 (CTLA4), mediates inhibitory immune responses, thus facilitating tumor immune evasion [6, 7, 8]. However, there have been successful cases of immunotherapy. Chimeric Antigen Receptor T‐cell (CAR‐T) therapy focuses on immune checkpoint inhibition, representing a significant advance in cancer treatment [9]. Moreover, clinical trials have shown favorable efficacy for both anti‐PD‐1 and anti‐CTLA‐4 treatments. Therefore, it is crucial to investigate the mechanisms underlying glioma‐related immunosuppression to improve the outcomes of glioma immunotherapy.

The APOBEC family consists of several enzymes, namely, Activation‐Induced Cytidine Deaminase (AICDA), APOBEC1, APOBEC2, APOBEC4, and seven homologs of the APOBEC3 subfamily [10]. These enzymes deaminate cytosine in single‐strand DNA (ssDNA) or mRNA, changing cytosine (C) into uracil (U) [11]. This process frequently leads to U–G mismatches, which may result in C > T point mutations at the replication fork. Moreover, the participation of uracil glycosylase in base excision repair and mismatch repair (MMR) can cause a high frequency of C > X mutations and chain breaks, possibly leading to chromosomal translocations. As a result, APOBEC activity can contribute to genomic instability. APOBEC also has a role in several biological processes, such as somatic hypermutation and class‐switch recombination during B‐cell maturation [12], the innate immune response against foreign retroviruses [13], and the epigenetic reprogramming of DNA demethylation [14].

Recent research has shown an increased expression of APOBEC in various tumors [15]. Mounting evidence indicates that APOBEC contributes to tumorigenesis in different organs [16]. The involvement of APOBEC extends beyond hematological malignancies to cover a range of solid tumors [17, 18]. It induces a high tumor mutation burden, promoting the development of new antigens that fuel tumor evolution and the potential for adaptive therapy. However, the role of APOBEC3C in glioma is still poorly understood. Our study found a correlation between APOBEC3C expression and the remodeling of the tumor microenvironment (TME) in glioma. This study examines the prognosis and therapeutic potential of APOBEC3C in glioma. Our findings help to clarify the genomic characteristics of glioma and may provide insights for future therapeutic strategies.

## 2 Materials and Methods

### 2.1 Data Acquisition and Download

In this study, 693 glioma patients were sourced from the mRNAseq_693 data set in the CGGA database [19], and 697 glioma patients were obtained from the TCGA database (Table 1) [20]. Additionally, the CGGA database offered sc‐RNA data for glioma patients, encompassing a total of 6148 cells from 73 biopsy samples collected via surgery from 14 patients. All data utilized in this study are publicly accessible.

**Table 1:** Clinical information of patients.

Characteristic	Subcategory	CGGA group total (%) (*N* = 693)		TCGA group total (%) (*N* = 697)	
		No.	%	No.	%
Age	Mean ± SD	43.3 ± 12.4	100.0	47.3 ± 15.3	100.00
Gender	Male	398	57.4	354	50.8
	Female	295	42.6	255	36.6
Grade	WHO II	188	27.1	216	31.0
	WHO III	255	36.8	241	34.6
	WHO IV	249	35.9	152	21.8
IDH_status	Wildtype	286	41.3	234	33.6
	Mutant	356	51.4	428	61.4
1p19q_status	Codel	145	20.9	169	24.2
	Noncodel	478	69.0	495	71.0
MGMT_status	Methylated	315	45.5	492	70.6
	Unmethylated	227	32.8	168	24.1
Subtype	Classical	83	12.0	90	12.9
	Neural	167	24.1	115	14.5
	Proneural	296	42.7	248	35.6
	Mesenchymal	147	21.2	105	15.1

*Note:* Number of patients enrolled in our study was listed.

### 2.2 Functional Enrichment Analysis

The gene set associated with APOBEC3C was subjected to Pearson's correlation analysis (*R* > 0.5, *p* < 0.001) and subsequently analyzed using the DAVID database (https://david.ncifcrf.gov). Functional enrichment analysis was conducted based on Gene Ontology (GO) and Kyoto Encyclopedia of Genes and Genomes (KEGG) pathways, with official gene symbols used as identifiers and *Homo sapiens* designated as the species. In this study, all enrichment results are ranked in ascending order according to their *p* values.

### 2.3 Gene Set Variation Analysis (GSVA)

We retrieved the immune response‐related gene set from AmiGO2 (http://amigo.geneontology.org/amigo). The relationship between APOBEC3C and the immune response was investigated using Pearson's correlation analysis, and a heatmap was generated using the Pheatmap package.

### 2.4 APOBEC3C and TME

To investigate the potential relationship between APOBEC3C and the glioma TME, we evaluated its correlation with immune cell populations using Pearson's correlation coefficient. Furthermore, the association between APOBEC3C and diverse immune‐infiltrating cell types was assessed through the TIMER database.

### 2.5 Single‐Cell Sequencing

To investigate the association between APOBEC3C and the TME in GBM, single‐cell RNA‐seq data were obtained from the CGGA database. The expression matrices from single‐cell sequencing were processed using the Seurat package in R. Gene expression data underwent normalization, followed by principal component analysis (PCA) using the “RunPCA” function. For data visualization, the “UMAP” package was employed, while cell annotation was performed using the “SingleR” package. The similarity of cell clusters was determined based on their characteristic genes, with higher gene similarity indicating greater cluster similarity.

### 2.6 Reagents and Antibodies

Antibodies targeting N‐cadherin, E‐cadherin, Vimentin, p65, and phosphorylated p65 (Ser536) were sourced from Cell Signaling Technology (Danvers, Massachusetts, USA). The anti‐GAPDH antibody was acquired from Protein Technology (Wuhan, China). Additionally, polyvinylidene fluoride (PVDF) membranes and chemiluminescent reagents were supplied by Millipore (Massachusetts, USA).

### 2.7 Cell Culture

The glioma cells were acquired from Wuhan Punosai Life Science and Technology Co Ltd. These cells were maintained in DMEM high‐glucose medium enriched with 10% fetal bovine serum (FBS) and a dual‐antibiotic solution. Cultivation was performed in a humidified incubator at 37°C with 5% CO_2_. The complete medium was replaced every 2–3 days, adjusted according to the proliferation rate of the cells.

### 2.8 Cell‐Scratch Assay

Following our previous method [21], well‐grown glioma cells were inoculated into 6‐well plates. When the cell growth density reached ~80%, each well was scratched horizontally with a 200 μL pipette tip. Subsequently, the cells were washed with PBS buffer. Finally, siRNA transfection was carried out according to the instructions of the transfection reagents provided by Neofect (Beijing) Biotech Co. Ltd., and the cells were continuously incubated in the incubator. Images were captured at 0, 12, and 24 h to observe the changes in the scratch width.

### 2.9 Transwell Assays

The migratory and invasive properties of cells were evaluated using the Transwell assay. For the migration assay, transfected cells were placed in the upper chamber and maintained in serum‐free DMEM, while the lower chamber contained DMEM supplemented with 10% FBS. In the invasion assay, the upper chamber was pre‐coated with Matrigel. Following a 24‐h incubation period, the cells were fixed with 20% formaldehyde and stained with 0.5% crystal violet. Images were subsequently captured using an inverted microscope.

### 2.10 Western Blot Analysis

Following cell transfection, cellular proteins were extracted according to the manufacturer's protocol. Proteins were initially separated via electrophoresis and then transferred onto a PVDF membrane. The membrane was blocked with skimmed milk at room temperature for 2 h. After blocking, the membrane was incubated with the primary antibody overnight at 4°C. Subsequently, the membrane was treated with the secondary antibody for 2 h, and protein bands were visualized using a chemiluminescent detection system.

### 2.11 Statistical Analysis

All data analyses and graphical visualizations were conducted using R software (version 4.3.1) and GraphPad. The *t*‐test was employed to evaluate the significance of differences between two sample means, while analysis of variance (ANOVA) was utilized to assess the significance of differences among more sample means. The log‐rank test was applied to evaluate the significance of prognostic indicators. Multivariate analysis was conducted using Cox regression models to identify independent prognostic factors in glioma patients. In all statistical tests, a *p* < 0.05 was considered statistically significant.

## 3 Results

### 3.1 Differential Expression of APOBEC3C in Pan‐Cancer

**Figure 1:**
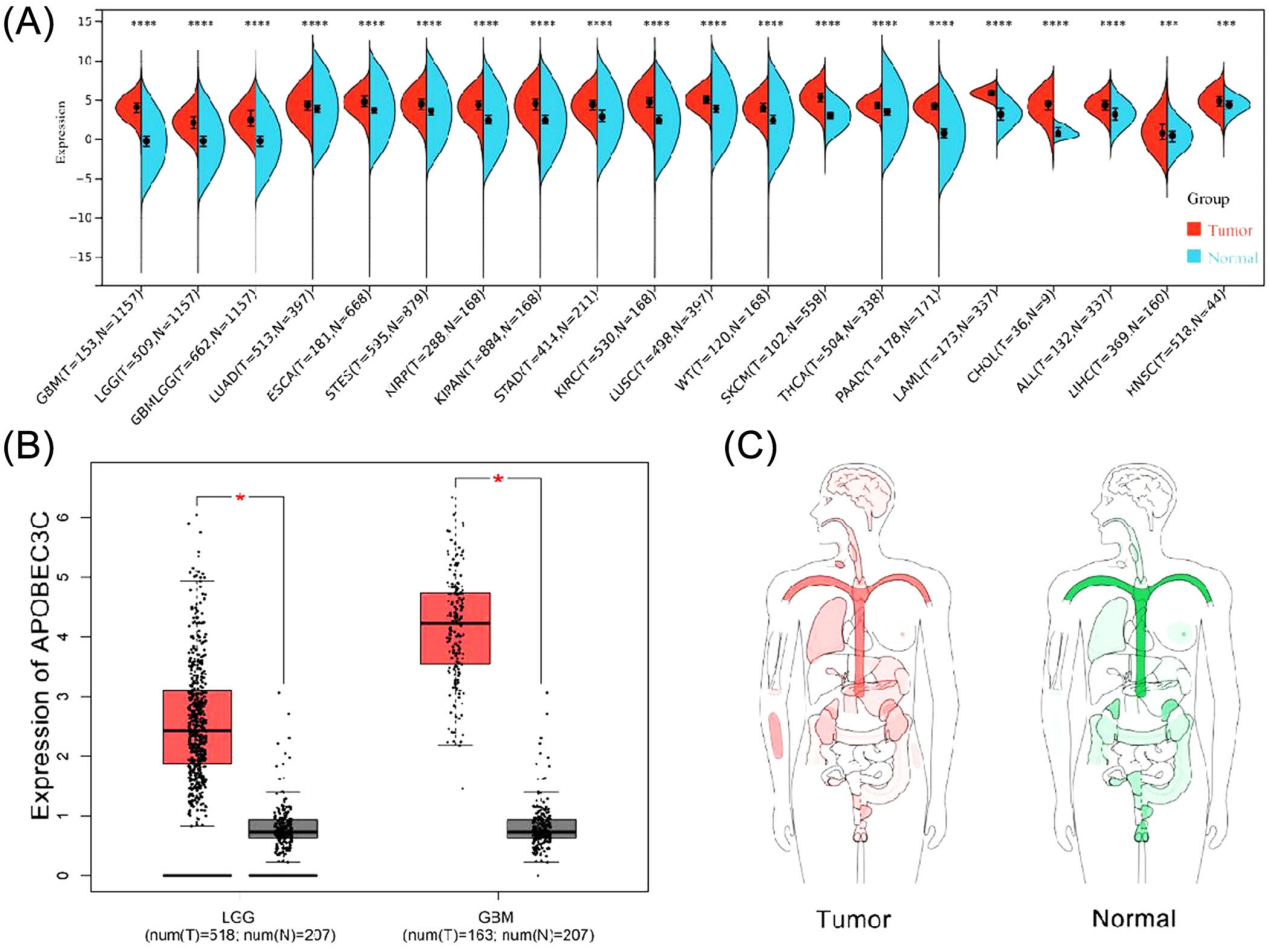
Differential expression of the APOBEC3C gene. (A) Differential expression analysis of APOBEC3C in pan‐cancer. (B) Levels of APOBEC3C expression in normal samples and gliomas of different WHO grades. (C) Differential expression of APOBEC3C in normal and tumor tissues in humans. The significance of the difference was tested using an unpaired *t*‐test. *p < 0.05; **p < 0.01; ***p < 0.001.

The TCGA database presents the APOBEC3C protein expression levels in 20 different types of cancer, suggesting that the expression of APOBEC3C in tumor samples is significantly higher than that in normal samples (Figure 1A). The GEPIA database (http://gepia.cancer-pku.cn/) further highlights the difference in APOBEC3C expression between normal tissues and tumor patients (Figure 1B,C).

### 3.2 APOBEC3C Is Enriched in Glioma Patients With Different Clinical Features and Molecular Differences

**Figure 2:**
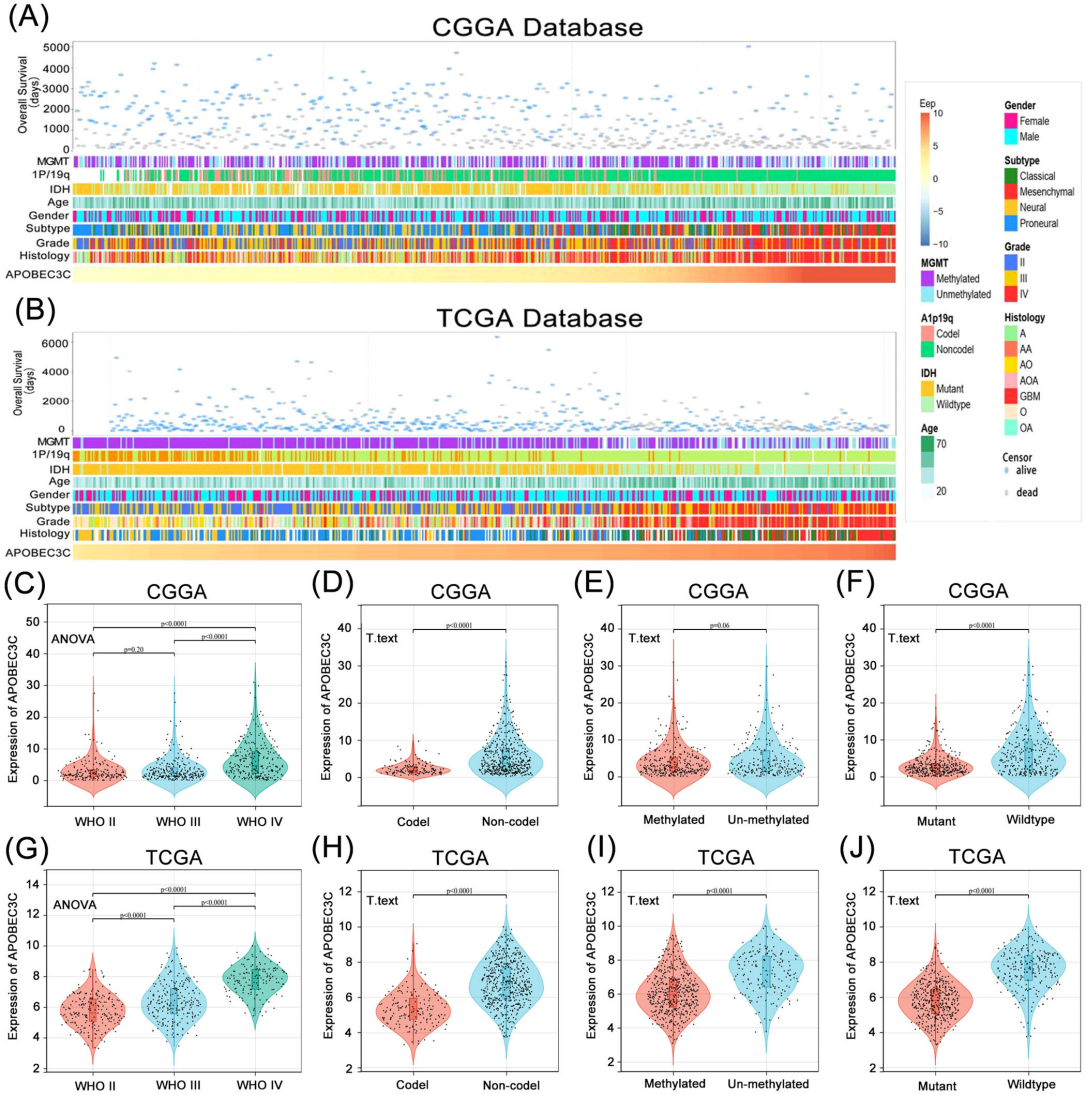
Association of APOBEC3C with clinical and molecular pathologic features of glioma patients. (A) Overview of glioma APOBEC3C‐related clinicopathologic features in CGGA. (B) Overview of glioma APOBEC3C‐related clinicopathologic features in TCGA. (C and G) In CGGA and TCGA, APOBEC3C expression was significantly higher in high‐grade gliomas. (D and H) A higher level of APOBEC3C expression is observed in CGGA and TCGA gliomas without a co‐deletion of 1p/19q. (E and I) O6‐methylguanine‐DNA methyltransferase (MGMT) promoter‐unmethylated gliomas express more APOBEC3C. (F and J) In CGGA and TCGA, APOBEC3C expression was increased in gliomas without mutations in IDH. The significance of the difference was tested using an unpaired *t*‐test and ANOVA.

We observed that patients with varying levels of APOBEC3C expression exhibited distinct clinical and molecular pathological features, such as WHO grade, 1p/19q co‐deletion status, MGMT promoter methylation status, and IDH mutation status. In both the CGGA and TCGA databases, the distribution of APOBEC3C expression levels displayed an asymmetric pattern (Figure 2A,B). Analysis of the CGGA database revealed that APOBEC3C expression was positively correlated with glioma WHO grade (Figure 2C), elevated in samples lacking 1p/19q codeletion (Figure 2D), and higher in IDH wild‐type patients (Figure 2F). These results were corroborated in the TCGA database (Figure 2G,H,J). Additionally, APOBEC3C expression was significantly increased in samples without MGMT promoter methylation in the TCGA database (Figure 2I), with a similar trend observed in the CGGA database (Figure 2E). Collectively, these findings suggest that gliomas with more aggressive phenotypes are characterized by elevated APOBEC3C expression levels.

### 3.3 An APOBEC3C Biomarker May Help Diagnose Glioma Mesenchymal Subtypes

**Figure 3:**
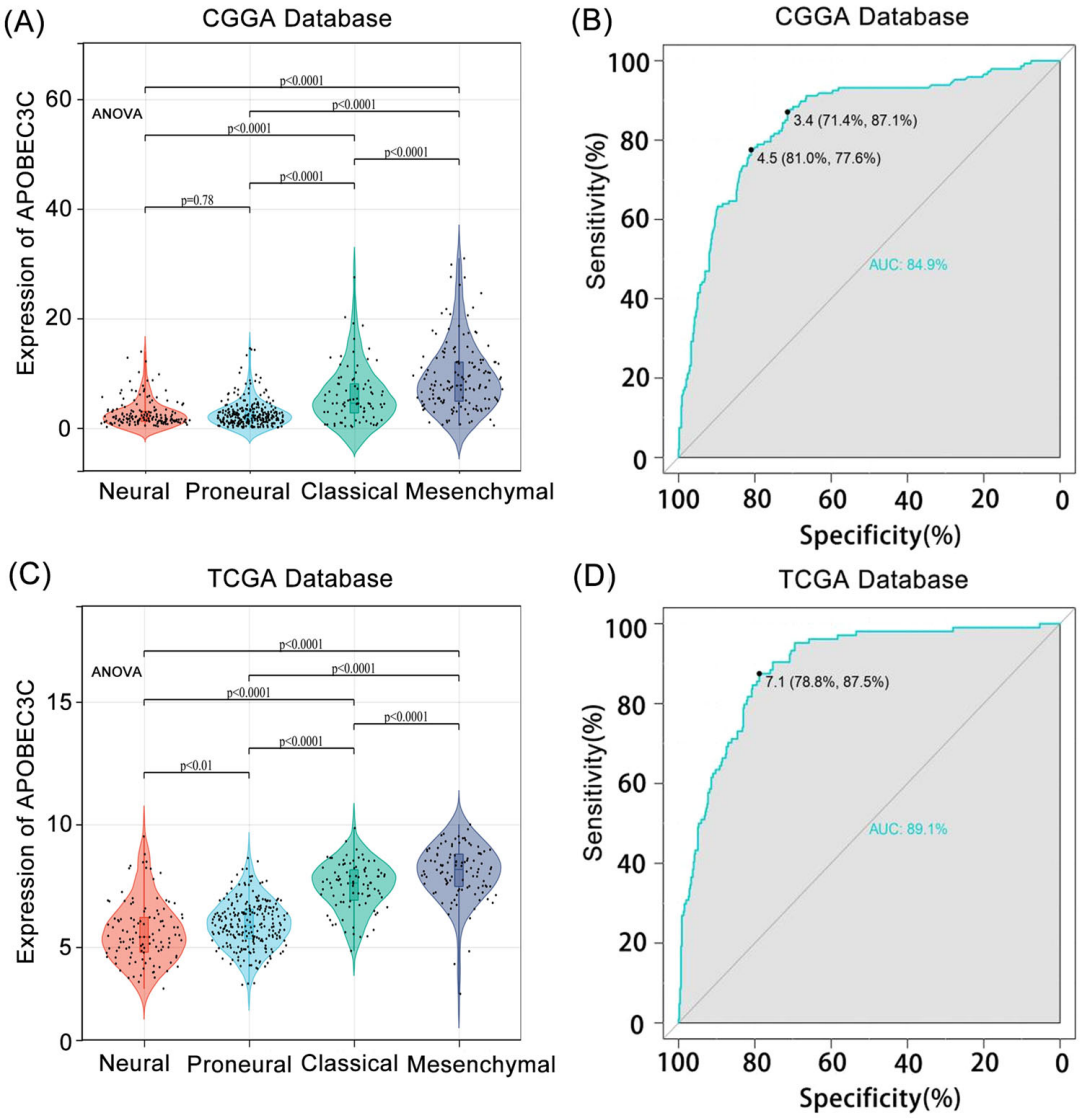
APOBEC3C is differentially expressed in histologic subtypes of gliomas. (A and C) APOBEC3C is enriched in glioma mesenchymal subtypes. (B and D) The ROC curve showed that APOBEC3C was highly specific in the mesenchymal subtype of glioma. Significance of differences was tested using ANOVA.

We explored the levels of APOBEC3C expression among different histological subtypes. In both the CGGA and TCGA databases, APOBEC3C was significantly overrepresented in mesenchymal cells (*p* < 0.05) (Figure 3A,C). To evaluate the specificity of APOBEC3C expression in glioma mesenchymal subtypes, we utilized receiver operating characteristic (ROC) curves. The analysis showed an AUC of 84.9% in the CGGA database (Figure 3B) and an AUC of 89.1% in the TCGA database (Figure 3D). APOBEC3C is preferentially enriched in the mesenchymal subtype with a poor prognosis, indicating its potential as a mesenchymal biomarker for gliomas. Since the mesenchymal subtype of GBM is more aggressive than other subtypes, these findings further confirm that APOBEC3C is positively correlated with the malignancy level of gliomas.

### 3.4 Glioma Immune and Inflammatory Responses Are Regulated by APOBEC3C

**Figure 4:**
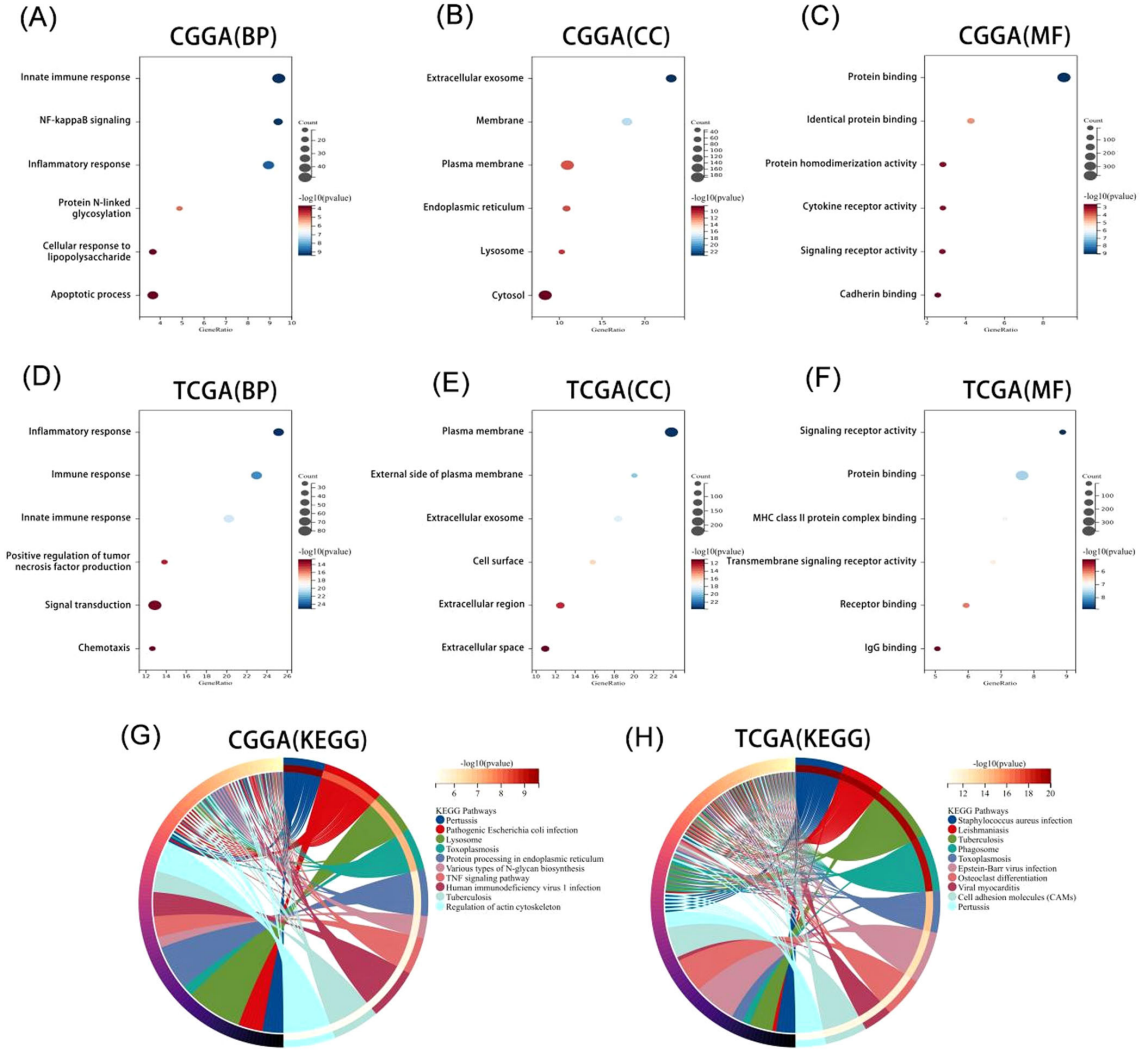
APOBEC3C is closely associated with immune and inflammatory responses. (A–C) APOBEC3C enriched biological processes (BP), cellular components (CC), and molecular functions (MF) in the CGGA database. (D–F) APOBEC3C enriched biological processes (BP), cellular components (CC), and molecular functions (MF) in the TCGA database. (G and H) KEGG analysis of the APOBEC3C.

To clarify the biological functions of APOBEC3C, we carried out Pearson's correlation analysis to identify genes most closely related to APOBEC3C. Subsequently, these gene sets were subjected to GO and KEGG analyses. The biological processes most closely related to APOBEC3C include the innate immune response, positive regulation of NF‐κB signaling, and inflammatory response in CGGA (Figure 4A). Moreover, APOBEC3C is located in exosomes and membranes (Figure 4B). Its molecular functions mainly involve protein binding, cytokine receptor activity, and signaling receptor activity, among others (Figure 4C). The most relevant signaling pathways are the lysosome and tumor necrosis factor signaling pathways (Figure 4G). These findings were reproduced in TCGA (Figure 4D–F,H). So, these data imply that APOBEC3C may play a significant role in glioma immune and inflammatory responses, making it an important immune target.

### 3.5 APOBEC3C Is Positively Correlated With Glioma‐Related Immune Pathways

**Figure 5:**
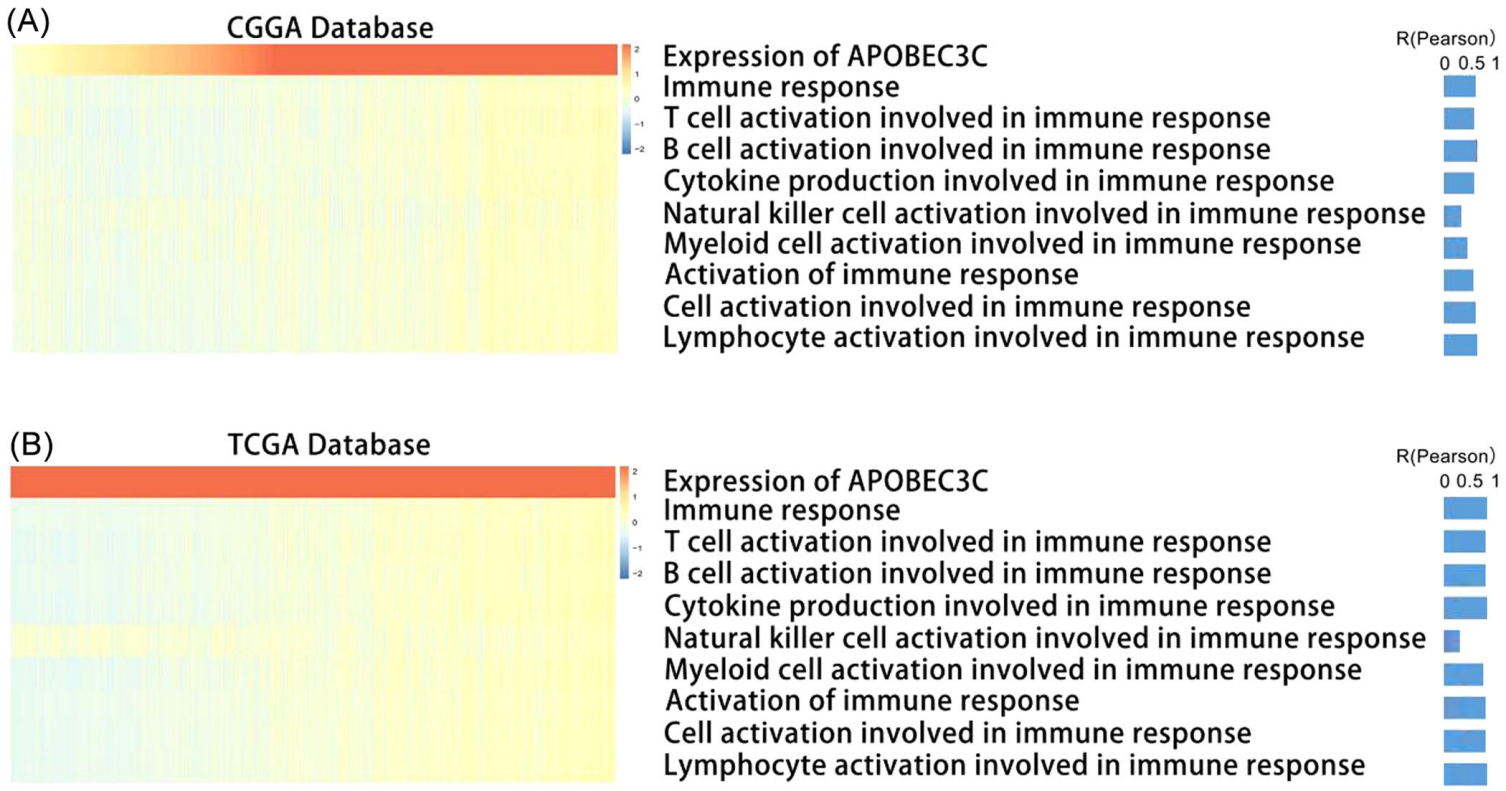
Correlation between APOBEC3C and immune function. (A and B) The APOBEC3C expression and immune function enrichment scores of each patient in CGGA and TCGA are shown in heatmaps sorted by APOBEC3C expression in ascending order.

In the context of immunogenic cancer cell death, lymphocytes are activated and chemokines and cytokines are released, which leads to apoptosis or necrosis [22]. Therefore, we explored the impact of APOBEC3C activation on immune responses. GSVA was carried out on the public databases to determine the immune process enrichment scores. A heatmap of the correlation analysis between the enrichment scores and APOBEC3C expression showed a positive correlation between most of the immune responses and APOBEC3C expression (Figure 5A). The results from the TCGA database confirmed these findings (Figure 5B). These data indicate a potential link between APOBEC3C and glioma immune responses.

### 3.6 APOBEC3C Is Associated With Immune Checkpoint Inhibitors and Inflammatory Activity

**Figure 6:**
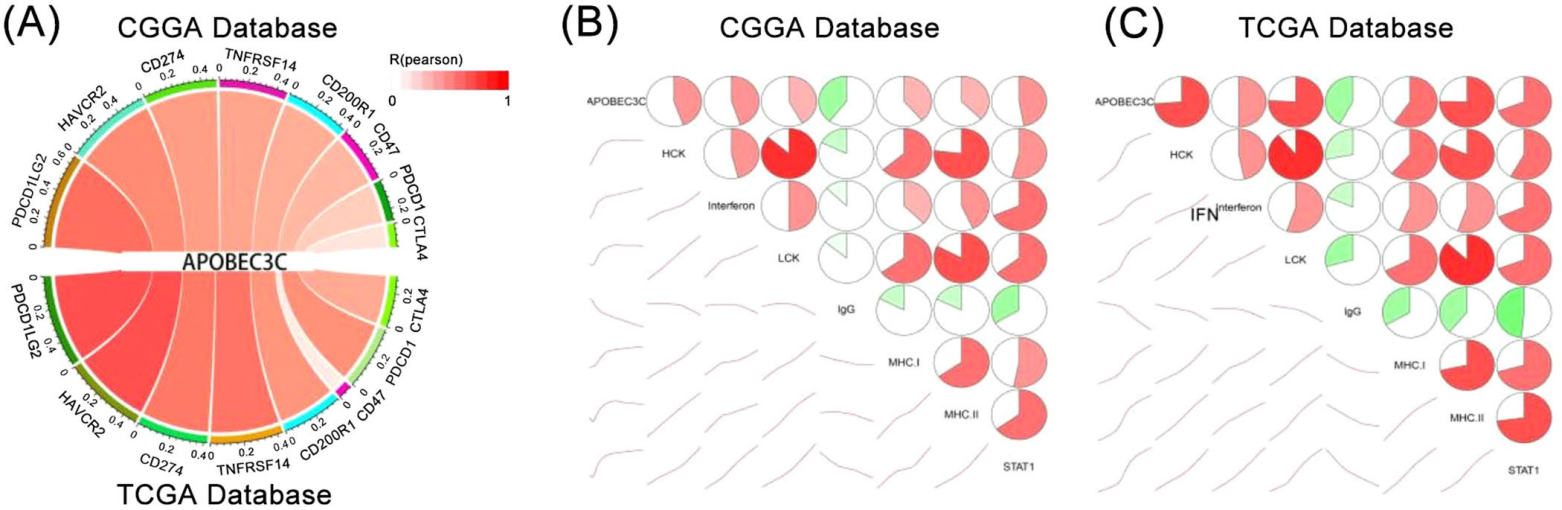
Correlation of APOBEC3C with immune checkpoints and inflammatory activity. (A) Correlation between APOBEC3C and inhibitory immune checkpoints. The width of the bands represents the *R* value, and the *p* values are all < 0.05. (B and C) APOBEC3C and inflammation‐related gene clusters. The lower left corner shows the correlation coefficient trend. Two‐color gradients and circle sizes are proportional to correlation. A positive correlation is represented by the red part and a negative correlation by the green part. The correlation was tested by Pearson's correlation analysis.

We found that APOBEC3C is involved in immune responses and closely related to inflammatory activity in the glioma TME. Using the CGGA and TCGA databases, we explored the correlation between APOBEC3C and inhibitory immune checkpoints. We randomly selected common ones such as PDCD1LG2, HAVCR2, CD274, TNFRSF14, CD200R1, CD47, PDCD1, and CTLA4. These checkpoints were strongly positively correlated with APOBEC3C (Figure 6A), indicating that APOBEC3C may suppress immune responses against gliomas.

To further investigate APOBEC3C‐related inflammatory activities, we identified 7 gene clusters consisting of 104 genes that represent various inflammatory and immune responses [23]. Correlation matrix plots were used to show the correlation between APOBEC3C and the seven gene clusters in both databases. The results indicated that APOBEC3C expression was correlated with most of the inflammatory responses, with the exception of the immunoglobulin G (IgG) metagenome, which was negatively correlated with APOBEC3C (Figure 6B,C).

### 3.7 The Relationship Between APOBEC3C and Immune Cell Infiltration in Gliomas

**Figure 7:**
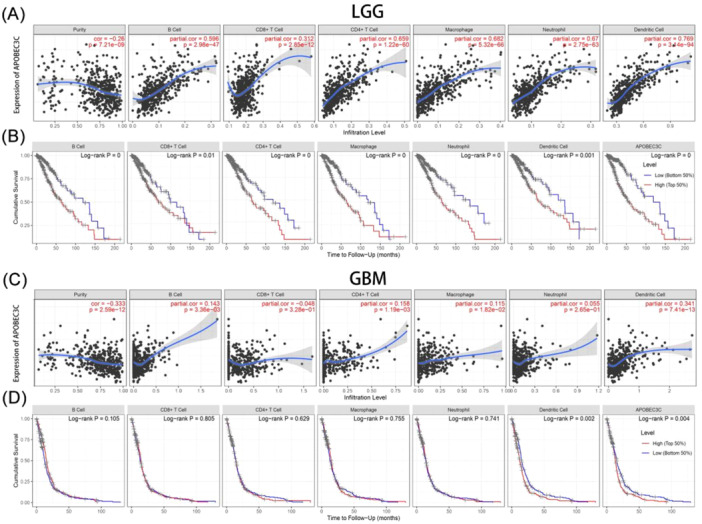
Correlation between APOBEC3C and immune infiltrating cells in TME. (A and C) LGG and GBM immune cell infiltration are associated with APOBEC3C. (B and D) Relationship between immune cell infiltration and cumulative survival in LGG and GBM.

To clarify the mechanism by which high APOBEC3C expression is associated with a poor prognosis in gliomas, the TIMER was used to reveal the association between APOBEC3C and the infiltration levels of six immune cell subtypes. In low‐grade gliomas (LGG), APOBEC3C expression was positively correlated with B cells (Cor = 0.596, *p* = 2.98E−47), CD8^+^ T cells (Cor = 0.312, *p* = 2.85E−12), CD4^+^ T cells (Cor = 0.659, *p* = 1.22E−60), macrophages (Cor = 0.682, *p* = 5.32E−66), dendritic cells (Cor = 0.769, *p* = 3.14E−94), and neutrophils (Cor = 0.67, *p* = 2.75E−63) (Figure 7A). Notably, patients with high levels of immune cell infiltration had a lower cumulative survival rate (Figure 7B). However, in GBM, immune cells were less associated with APOBEC3C (Figure 7C,D). Remarkably, there was no significant correlation between APOBEC3C expression and CD8^+^ T‐cell infiltration levels in both LGG and GBM, and a clear negative correlation was observed in GBM patients (Figure 7A,C). High expression of APOBEC3C may lead to an increase in macrophages and dendritic cells while decreasing CD8^+^ T cells, thus contributing to the poor cumulative survival rate of glioma patients (Figure 7B). Tumor‐infiltrating lymphocytes, such as tumor‐associated macrophages (TAMs) and tumor‐infiltrating neutrophils, can affect the efficacy of chemotherapy and immunotherapy [24]. APOBEC3C may inhibit the antitumor immune process mediated by T cells by activating macrophages and dendritic cells.

### 3.8 Relationship Between APOBEC3C and Markers of Immune Infiltration in Gliomas

**Figure 8:**
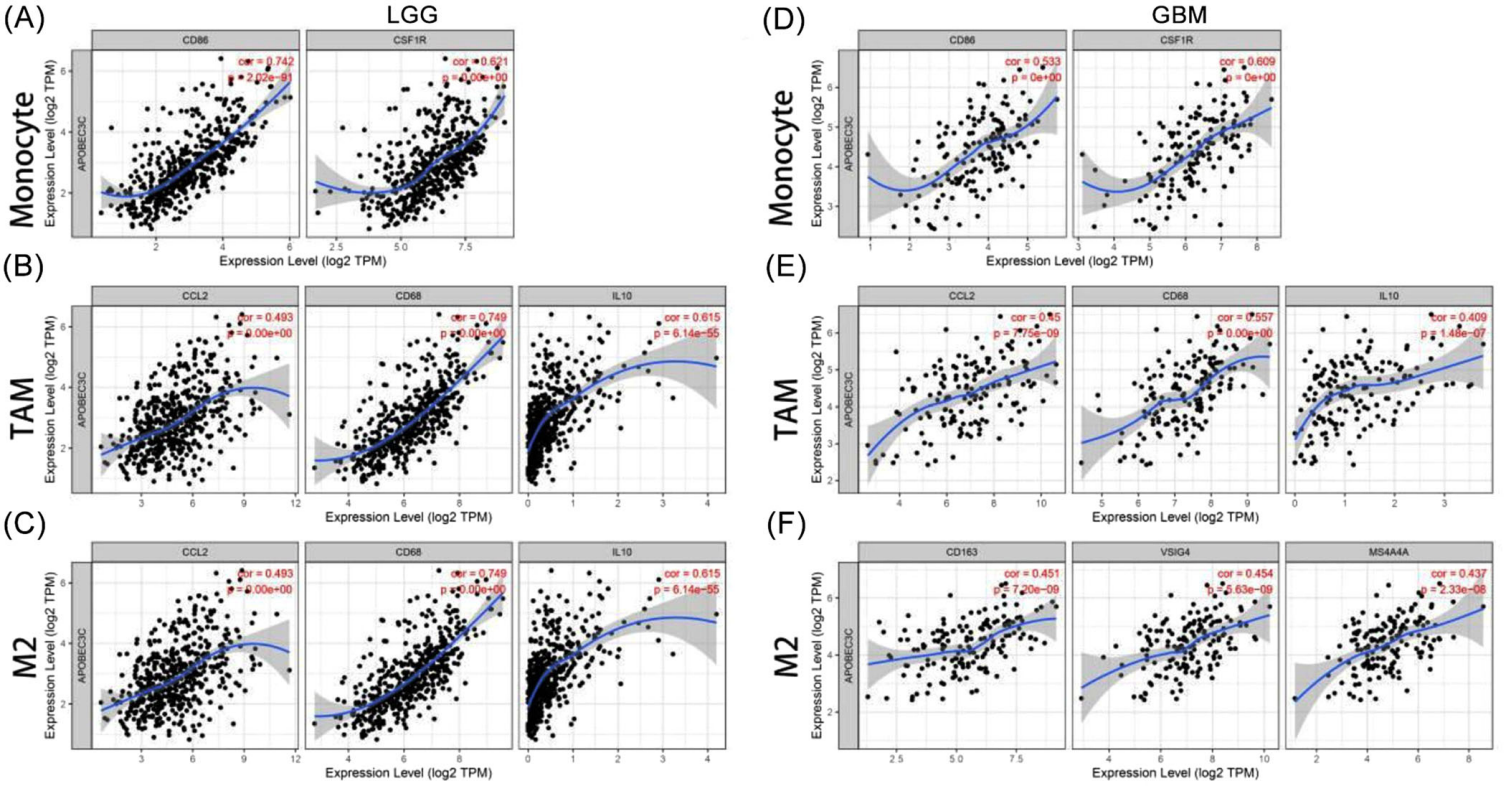
The expression of APOBEC3C correlates with that of marker genes for monocytes (A and D), tumor‐associated macrophages (B and E), and M2 macrophages (C and F).

Infiltrating immune cells in the TME influence the biological behavior of tumors and patient outcomes. The TIMER was used to explore the correlation between APOBEC3C and multiple immune cell markers. Gliomas with APOBEC3C expression presented significant correlations with various immune markers in different immune cells and T‐cell subsets (Table 2). In gliomas, APOBEC3C was positively correlated with markers of monocytes (Figure 8A,D), TAMs (Figure 8B,E), and M2 macrophages (Figure 8C,F). APOBEC3C is involved in regulating macrophage polarization in gliomas. Moreover, APOBEC3C has been associated with several markers of regulatory T cells (Tregs) and T‐cell exhaustion, such as TIM‐3. Depletion of Tregs and T cells is crucial for tumor immune evasion. Therefore, APOBEC3C may play an immunomodulatory role in gliomas. Significantly, T‐cell immunoglobulin mucin 3 (TIM‐3), a key gene regulating T‐cell exhaustion, was positively correlated with APOBEC3C expression. This shows that high APOBEC3C expression is related to TIM‐3‐mediated T‐cell exhaustion. Furthermore, it was confirmed that APOBEC3C is specifically associated with infiltrating immune cells in gliomas, suggesting that APOBEC3C plays a vital role in the immune evasion mechanism of gliomas.

**Table 2:** Correlation of APOBEC3C expression with immune cell‐related genes and markers in TIMER.

Description	Gene markers	LGG		GBM	
		Cor	*p*	Cor	*p*
Monocyte	CD86	0.742	***	0.533	***
	CD115 (CSF1R)	0.621	***	0.609	***
TAM	CCL2	0.493	***	0.450	***
	CD68	0.749	***	0.557	***
	IL10	0.615	***	0.409	***
M1 macrophage	INOS (NOS2)	−0.113	*	0.133	0.101
	IRF5	0.698	***	0.507	***
	COX2 (PTGS2)	0.134	**	0.287	***
M2 macrophage	CD163	0.494	***	0.451	***
	VSIG4	0.612	***	0.454	***
	MS4A4A	0.578	***	0.437	***
Dendritic cell	HLA‐DPB1	0.682	***	0.544	***
	HLA‐DQB1	0.565	***	0.298	***
	HLA‐DRA	0.731	***	0.504	***
	HLA‐DPA1	0.717	***	0.500	***
	BDCA‐1 (CD1C)	0.385	***	0.199	*
	BDCA‐4 (NRP1)	0.379	***	0.460	***
	CD11c (ITGAX)	0.562	***	0.317	***
Treg	FOXP3	0.015	0.735	0.367	***
	CCR8	0.181	***	0.333	***
	STAT5B	0.141	*	−0.070	0.392
T‐cell exhaustion	PD‐1 (PDCD1)	0.514	***	0.196	*
	CTLA4	0.345	***	0.345	***
	LAG3	0.193	***	0.007	0.936
	TIM‐3 (HAVCR2)	0.742	***	0.493	***
	GZMB	0.273	***	0.273	***
T‐cell (general)	CD3D	0.509	***	0.347	***
	CD3E	0.560	***	0.420	***
	CD2	0.575	***	0.435	***
CD8^+^ T‐cell	CD8A	0.242	***	0.118	0.147
	CD8B	0.260	***	0.205	*
B‐cell	CD19	0.396	***	0.159	0.050
	CD79A	0.262	***	0.011	0.894
Neutrophils	CD66b (CEACAM8)	−0.022	0.623	−0.091	0.261
	CD11b (ITGAM)	0.686	***	0.565	***
	CCR7	0.403	***	0.335	***
CD4^+^ T‐cell	CD4	0.733	***	0.573	***

*p < 0.05; **p < 0.01; ***p < 0.001.

### 3.9 APOBEC3C Is Enriched in T Cell in GBM

**Figure 9:**
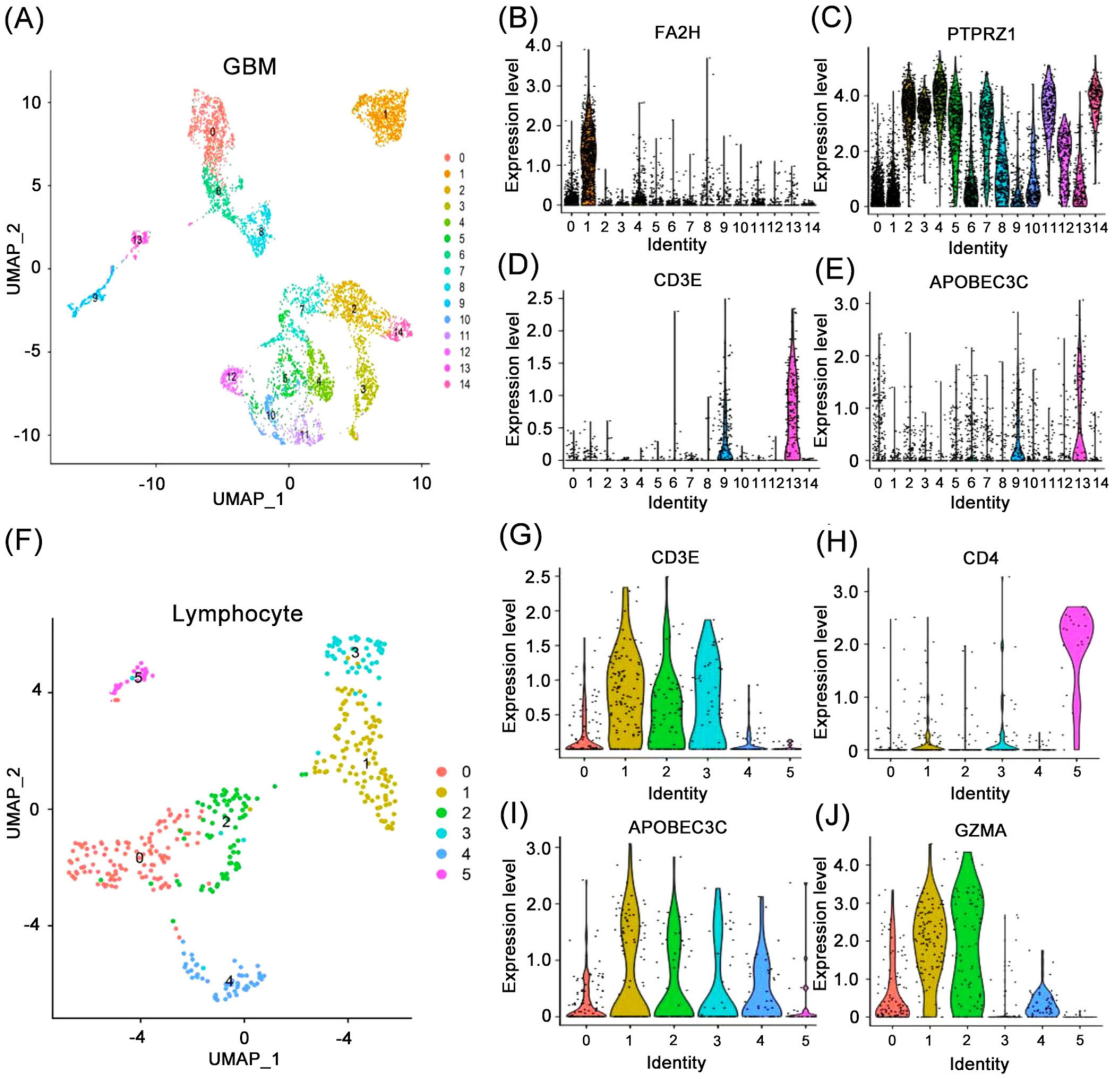
Expression model of APOBEC3C in GBM single‐cell sequencing. (A) Cell cluster of single‐cell sequencing data from GBM patients. (B–D) Expression of characteristic cell markers in GBM sample clusters. (E) Expression of APOBEC3C in GBM sample cluster. (F) GBM single‐cell sequencing data lymphocyte classification. (G–I) Expression of characteristic cell markers in lymphocyte subsets sample subsets. (J) Expression of APOBEC3C in lymphocyte subsets sample subsets.

APOBEC3C is associated with various immune‐infiltrating cell types in the glioma TME. We utilized single‐cell scRNA‐seq to analyze APOBEC3C expression levels in different cell clusters in GBM. Using GBM single‐cell sequencing data, the R software “UMAP” package segregated the GBM single‐cell sequencing data into 15 cell clusters and identified the differentially expressed genes of each cell cluster (Figure 9A). Based on the expression of characteristic markers, the 0th, 6th, and 8th cell clusters were characterized as macrophages, the 1st cell cluster as oligodendrocytes (Figure 9B), and the 9th and 13th cell clusters as T‐cells (Figure 9D). The remaining cell clusters were classified as tumor cells (Figure 9C). It was observed that APOBEC3C exhibited high expression in the 9th and 13th cell clusters designated as lymphocytes (Figure 9E). Subsequently, lymphocytes were isolated for further analysis, revealing that they could be further divided into 6 cell clusters (Figure 9F), with APOBEC3C exhibiting consistency with CD8^+^ T cell markers (Figure 9G,I), but not CD4^+^ T cell markers (Figure 9H). These findings confirm the previous hypothesis that APOBEC3C is mediated by CD8^+^ T cells in the TME of GBM patients, and further influences the development of tumor cells and worsens prognosis. Additionally, we discovered that cytotoxic genes (GZMA, GZMB, PRF1, and GNLY) were highly expressed in two of these cell clusters (Figure 9J).

### 3.10 The APOBEC3C Gene Is an Independent Prognostic Factor in the Survival of Patients With Gliomas

**Figure 10:**
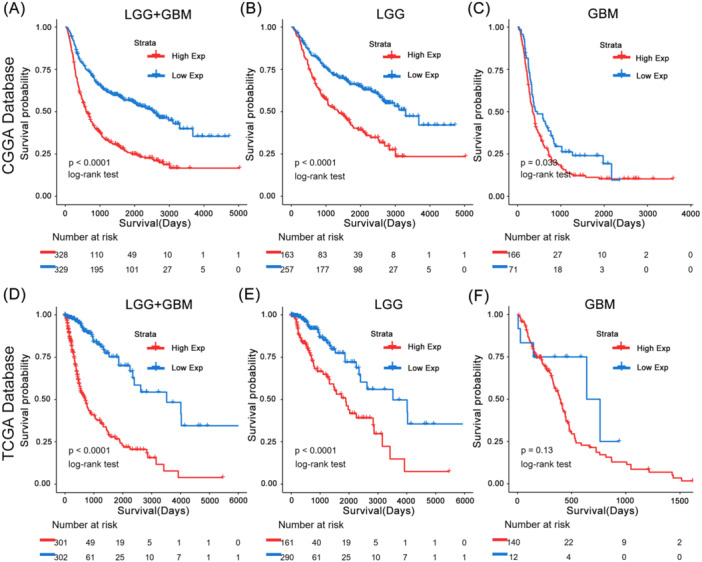
K–M survival curve of APOBEC3C expression in glioma patients. (A–C) Prognostic analysis of APOBEC3C differential expression in CGGA databases. (D–F) Prognostic analysis of APOBEC3C differential expression in TCGA databases. The significance of the prognostic value was tested by a log‐rank test.

Based on the CGGA and TCGA databases, we conducted Kaplan–Meier (K–M) and Cox proportional hazards model analyses to assess the prognostic significance of APOBEC3C in gliomas. Patients with high APOBEC3C expression exhibited significantly shorter OS compared to those with low expression in the CGGA database (Figure 10A). The TCGA database further confirmed the prognostic value of APOBEC3C (Figure 10D), consistently indicating a poor prognosis for patients with elevated APOBEC3C expression. To mitigate the impact of tumor grade, we reanalyzed the prognostic outcomes for patients with gliomas of varying grades (Figure 10B,C,E,F). Notably, APOBEC3C emerged as an independent prognostic factor in the Cox proportional hazards model analysis (Tables 3 and 4).

**Table 3:** An analysis of prognostic parameters in the CGGA database OS.

Variable	Univariate analysis		Multivariate analysis	
	HR (95% CI)	*p*	HR (95% CI)	*p*
Subtype	2.182 (1.785–2.667)	***	2.450 (1.917–3.130)	***
WHO grade (II vs. III)	0.144 (0.105–0.197)	***	0.215 (0.139–0.333)	***
WHO grade (II vs. IV)	0.365 (0.293–0.455)	***	0.525 (0.386–0.714)	***
Age	1.026 (1.018–1.035)	***	1.010 (1.000–1.019)	*
IDH status	3.092 (2.509–3.81)	***	1.778 (1.298–2.434)	***
1p/19q codel	3.731 (2.690–5.175)	***	2.627 (1.750–3.945)	***
MGMT status	1.257 (1.010–1.566)	*	1.122 (0.881–1.427)	0.351
APOBEC3C	1.057 (1.041–1.074)	***	0.970 (0.946–0.994)	*

*p < 0.05; ***p < 0.001.

**Table 4:** An analysis of prognostic parameters in the TCGA database OS.

Variable	Univariate analysis		Multivariate analysis	
	HR (95% CI)	*p*	HR (95% CI)	*p*
WHO grade (II vs. III)	0.050 (0.030–0.082)	***	0.349 (0.179–0.680)	**
WHO grade (II vs. IV)	0.162 (0.114–0.232)	***	0.700 (0.444–1.102)	0.123
Age	1.075 (1.063–1.088)	***	1.054 (1.038–1.070)	***
IDH status	11.046 (7.754–15.735)	***	2.166 (1.212–3.871)	**
1p/19q codel	4.538 (2.670–7.714)	***	1.568 (0.828–2.969)	0.168
MGMT status	3.205 (2.311–4.445)	***	1.144 (0.782–1.675)	0.488
APOBEC3C	2.243 (1.972–2.551)	***	1.506 (1.265–1.792)	***

**p < 0.01; ***p < 0.001.

### 3.11 APOBEC3C Inhibits the Malignant Progression of Glioma via the NF‐κB Signaling Pathway

**Figure 11:**
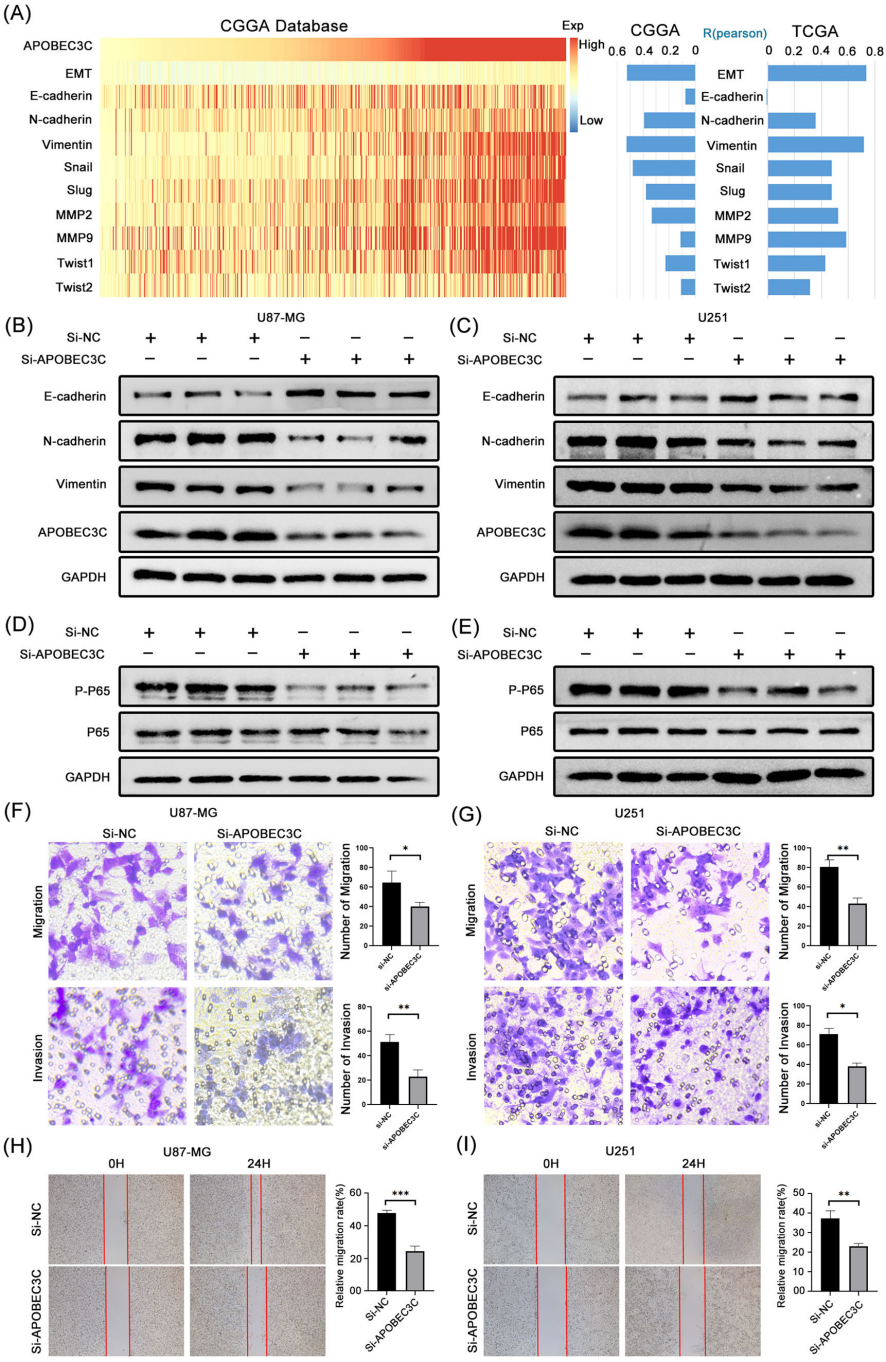
APOBEC3C promotes malignant progression in gliomas. (A) APOBEC3C and EMT correlation analysis. (B and C) APOBEC3C regulates the expression level of EMT‐related proteins. (D and E) AOBEC3C inhibits the activation of NF‐κB signaling pathway. (F and G) Transwell assay. (H and I) Cell‐scratch assay. *p < 0.05; **p < 0.01; ***p < 0.001.

To validate the effect of APOBEC3C on the malignant progression of gliomas, we analyzed the correlation between APOBEC3C and the EMT process as well as its representative genes. It was found that APOBEC3C had a significant correlation with the EMT process (Figure 11A). Furthermore, transwell experiments demonstrated that knocking down APOBEC3C using siRNA technology under in‐vitro conditions could significantly inhibit the migration and invasion abilities of glioma cells (Figure 11F). Similarly, cell‐scratch experiments showed that knocking down APOBEC3C could inhibit the horizontal migration ability of glioma cells U87‐MG (Figure 11H). Further western blot experiments indicated that knocking down APOBEC3C could regulate the expression levels of EMT‐related proteins (Figure 11B). Finally, to verify the results of GO enrichment analysis, we investigated the effect of APOBEC3C on the common NF‐κB signaling pathway. Western blot results showed that knocking down APOBEC3C could significantly inhibit the activation of the NF‐κB signaling pathway, thus exerting antitumor effects (Figure 11D). Not only that, but we also obtained similar results when using the U251 glioma cell line (Figure 11C,E,G,I).

## 4 Discussion

The TME is a hot topic in current tumor research. Drug resistance in gliomas is closely related to their unique metabolic mechanisms and the complex immunosuppressive microenvironment in which they exist [25]. Even with a combination of surgery and radiotherapy, the prognosis of glioma patients cannot be significantly improved. Therefore, the development and validation of more effective tumor immunomarkers are of great significance in improving the overall prognosis of glioma patients.

The APOBEC3 family has been identified as an endogenous source of somatic mutations associated with diseases such as cancer and also plays a crucial role in protecting cells from endogenous and exogenous DNA damage. It has been found that the deletion of APOBEC3B affects the neoantigen load and immune cell composition in BRCA patients. Additionally, APOBEC3 mutations are frequently observed in non‐small‐cell lung cancer (NSCLC) patients who have improved clinical symptoms after long‐term immunotherapy. In NSCLC, elevated APOBEC3 expression is associated with a poor prognosis [26]. Previous studies have demonstrated that APOBEC3 contributes to subclonal diversity and cancer heterogeneity in certain cancers. In breast cancer, the upregulation of APOBEC3s (especially APOBEC3B) leads to elevated TP53 and PIK3CA mutations [27]. Moreover, elevated APOBEC3G levels make cells more susceptible to cisplatin, thus potentially improving the prognosis of patients with head and neck squamous cell carcinoma (HNSCC) [28]. Recent research reports have also demonstrated the potential value of APOBEC3C in glioma patients [29, 30].

Glioma cells are capable of secreting various immunomodulatory factors and attracting a variety of cells, including immune cells, into the TME. A previous study showed that different levels of immune cells in gliomas were related to prognosis [31]. Glioma‐associated macrophages, microglia, dendritic cells, and neutrophils together form the glioma TME, which monitors and suppresses the tumor immune response [32]. In the present study, APOBEC3C was identified as a new prognostic biomarker. It showed a positive correlation with the abundance of immune cell infiltration and cell markers such as monocytes, TAM, M2 macrophages, and dendritic cells in gliomas. In addition, APOBEC3C was found to be highly expressed in CD8^+^ T cells. Therefore, differences in immune cell content may potentially be an important factor affecting the different survival rates seen among various tumor types.

Macrophages, a prominent cell type within the immune system, act as crucial mediators of inflammation [33]. During the process of tumor progression, macrophages create an inflammatory environment, promote angiogenesis, facilitate tumor cell migration and invasion, and hinder antitumor immune responses [34]. Previous studies have emphasized the correlation between the accumulation of TAM and adverse clinical outcomes [35]. Dendritic cells, which function as antigen‐presenting cells, coordinate T‐cell immune responses and play a crucial role in initiating tumor immunity [36]. The activation of tumor immunity highly depends on mature dendritic cells providing costimulatory signals to T cells; however, this is often insufficient for eliciting effective immune responses against tumors on its own [37]. Tumor cells can utilize dendritic cells to evade immune surveillance in the cancerous environment. B cells are essential components of the human immune system, participating in crucial biological functions such as antigen presentation, T‐cell activation, and antibody production. B cells secrete cytokines that are involved in tumor promotion [38]. Neutrophils, as key effector cells in the immune system, have more ambiguous roles in tumor pathogenesis compared to other immune cells. Although they have been traditionally regarded as mounting a defensive response against tumor cells, emerging evidence indicates that tumor‐associated neutrophils are associated with tumor progression [39]. Various clinical studies have demonstrated the usefulness of neutrophil count as a prognostic biomarker for different types of cancers [40].

Our study disclosed a correlation between the expression of APOBEC3C and diverse immune cells as well as their markers. Moreover, APOBEC3C was highly expressed in malignant glioma subtypes and could function as a biomarker for mesenchymal glioma subtypes. In line with previous research, knocking down APOBEC3C was capable of inhibiting the malignant progression of gliomas via the NF‐κB signaling pathway [41]. The results of in‐vitro experiments also attested to the efficacy of APOBEC3C as a potential therapeutic target for glioma.

Although the results of this study enhance our understanding of the relationship between APOBEC3C and gliomas, several limitations remain. First, our bioinformatics analysis was based solely on the CGGA and TCGA databases, and we did not conduct animal experiments for validation. Second, the limited sample size in public databases, along with the inherent heterogeneity of the data, may lead to gaps in data processing and analysis. Third, while retrospective studies can be conducted quickly to analyze results, additional prospective studies are necessary to mitigate analytical bias. Finally, the mechanism of action of APOBEC3C in gliomas requires further exploration for a deeper understanding.

Immunotherapy is an important research direction in the field of oncology. Future research should focus on elucidating the role of APOBEC3C in glioma through integrated single‐cell sequencing and multiomics analysis to uncover its interactions with immune cells in the TME, followed by experimental validation of its impact on immunotherapy efficacy. Large‐scale clinical cohort studies are needed to assess its prognostic value across glioma subtypes, and mechanistic investigations should explore APOBEC3C's involvement in immune evasion, ultimately paving the way for clinical translation of APOBEC3C‐targeted therapies to enhance immunotherapy outcomes.

## 5 Conclusion

The results of this study suggest that high expression of APOBEC3C is associated with poor prognosis in glioma and may affect immune regulation. Knocking down APOBEC3C can inhibit the invasion and migration abilities of glioma cells. In addition, APOBEC3C can regulate the activation of the NF‐κB signaling pathway, which is expected to be a target for improving immunotherapy in glioma patients.

## Author Contributions

**Chao Zhang:** conceptualization, data curation, formal analysis, investigation, software, validation, visualization, writing – original draft, writing – review and editing. **Tao Yang:** conceptualization, data curation, formal analysis, resources, software, validation, visualization, writing – original draft. **Yuhang Tang:** conceptualization, data curation, formal analysis, resources, writing – original draft, writing – review and editing. **Dong Yu:** conceptualization, resources, software, supervision, validation, visualization. **Shiqiang Hou:** data curation, funding acquisition, resources, software, validation, visualization. **Ning Lin:** funding acquisition, project administration, resources, validation, visualization, writing – review and editing. **Qun Li:** funding acquisition, project administration, resources, validation, visualization, writing – review and editing.

## Conflicts of Interest

The authors declare no conflicts of interest.

## Data Availability

All the data sets used and analyzed during the current study are downloaded from CGGA (http://cancergenome.nih.gov/), TCGA (http://www.cgga.org.cn), DAVID (https://david.ncifcrf.gov/), and AmiGO2 (http://amigo.geneontology.org/amigo), which are available from the corresponding authors on reasonable request.
